# Retention in an mHealth App Aiming to Promote the Use of HIV Pre-Exposure Prophylaxis Among Female Sex Workers in Dar es Salaam, Tanzania: Prospective Cohort Study

**DOI:** 10.2196/46853

**Published:** 2023-10-17

**Authors:** Christopher H Mbotwa, Method R Kazaura, Kåre Moen, Melkizedeck T Leshabari, Emmy Metta, Elia J Mmbaga

**Affiliations:** 1 Department of Epidemiology and Biostatistics, Muhimbili University of Health and Allied Sciences, Dar es Salaam, United Republic of Tanzania; 2 Mbeya College of Health and Allied Sciences, University of Dar es Salaam, Mbeya, United Republic of Tanzania; 3 Department of Community Medicine and Global Health, Faculty of Medicine, University of Oslo, Oslo, Norway; 4 Department of Behavioural sciences, Muhimbili University of Health and Allied Sciences, Dar es Salaam, United Republic of Tanzania

**Keywords:** mobile health, retention, engagement, mHealth, sex workers, pre-exposure prophylaxis, HIV, Africa, PrEP

## Abstract

**Background:**

Increasing access to smartphones in sub-Saharan Africa offers an opportunity to leverage mobile health (mHealth) technology to improve access to health care in underserved populations. In the domain of HIV prevention, mHealth interventions can potentially contribute to solving the challenges of suboptimal adherence to pre-exposure prophylaxis (PrEP) and low retention in PrEP services among populations most vulnerable to HIV acquisition. However, there is a gap in the knowledge about the use of such interventions in sub-Saharan Africa.

**Objective:**

This study aims to evaluate the extent and predictors of retention in an mHealth app (*Jichunge*) that aims to promote adherence to PrEP and retention in PrEP care among female sex workers in Dar es Salaam, Tanzania.

**Methods:**

A prospective cohort of female sex workers residing in Dar es Salaam were recruited, using respondent-driven sampling. All participants were provided with the *Jichunge* app as they started PrEP. A questionnaire was used to collect data on sociodemographics and other structural factors, while app use data for the 60-day period following the first 150 days of being in the intervention arm were extracted from the app’s back end. A multivariable log-binomial model was used to determine predictors of 6-month retention in the *Jichunge* app.

**Results:**

A total of 470 female sex workers were recruited. Nearly three-quarters of participants (206/284, 72.5%) who came to the 6-month follow-up interview no longer had the *Jichunge* app on their phones. The majority of these participants (193/206, 93.7%) no longer had access to the app because of issues related to their phones. Data extracted from the back end of the app showed that the use of the app declined over time, and only 13.4% (63/470) of the participants were retained (continued to use the app) after 6 months of intervention. At 6 months, women aged ≥35 years were >2 times more likely to use the app than women aged 18 to 24 years (adjusted risk ratio [aRR] 2.2, 95% CI 1.2-4.1; *P*=.01). Furthermore, retention in the app was higher among participants who demonstrated high PrEP awareness at baseline (aRR 1.8, 95% CI 1.1-3; *P*=.01) and among those who had experienced financial difficulties due to health care spending (aRR 1.9, 95% CI 1.2-3.2; *P*=.01).

**Conclusions:**

Most female sex workers (206/284, 72.5%) who were enrolled in PrEP care in Tanzania no longer used the *Jichunge* app after 6 months. Retention in the app at 6 months was predicted by older age, high PrEP awareness, and financial difficulties due to health care spending. Strategies for the long-term retention of participants in mHealth apps, such as systems for reinstallations of apps, should be considered during the design phase.

## Introduction

Mobile health (mHealth) apps have increasingly been reported to be effective in promoting access and adherence to treatment for both communicable diseases and noncommunicable diseases globally [1]. The increased use of smartphones in low- and middle-income countries, such as many of those in sub-Saharan Africa, offers an opportunity to leverage mHealth technology for health promotion purposes [2]. mHealth can be useful in minimizing some structural barriers to health care access (eg, by reducing the impact of long distance to health care facilities and reducing the likelihood of stigma experiences associated with certain conditions and treatments) and hence potentially contributes to the achievement of universal health coverage [3,4].

In the domain of biomedical HIV prevention, mHealth interventions have been shown to improve the effectiveness of pre-exposure prophylaxis (PrEP; eg, in the United States) through supporting adherence to medication and retention in care [5-7]. Information on the use of mHealth for such purposes is limited in sub-Saharan Africa. Furthermore, there is a scarcity of evidence on the extent and predictors of the longer-term use of mHealth apps for HIV prevention in populations most vulnerable to HIV acquisition and transmission. The longer-term use of many mHealth apps is fundamental if the whole spectrum of their intended effects is to be realized. Therefore, understanding the long-term use of various types of mHealth apps, as well as the association between use and different types of users and user characteristics, is vital if one aims to develop successful and innovative interventions tailored to the specific needs of the targeted population. The use of any app can be influenced by individual and structural factors, as well as by the condition for which its use is intended. For example, Crafoord et al [8] found that older age and higher education predicted higher engagement with an interactive mHealth app among patients with breast cancer or prostate cancer in Sweden. On the other hand, a study by Nelson et al [9] found that the use of a text message intervention to support self-care among patients with type 2 diabetes in the United States did not differ by sociodemographic characteristics.

In this study, we focused on retention in an mHealth app that aims to support PrEP use among female sex workers in Tanzania. Female sex workers are disproportionately affected by HIV [10,11]. According to the UNAIDS (Joint United Nations Programme on HIV/AIDS), the risk of acquiring HIV is 30 times higher among female sex workers than that among adult women in general [10]. In Tanzania, a recent study estimated the HIV prevalence among female sex workers in Dar es Salaam to be 15.3% [12], which is 3 times higher than the HIV prevalence in the general population [13]. In all African countries except Senegal, sex work is criminalized and not regulated by health policies [14], and this increases female sex workers’ vulnerability to stigma, HIV infection, and a lack of adequate access to health services [15,16]. Thus, interventions that may do away with some barriers to health care service access among female sex workers could potentially make significant contributions toward achieving the global goal of ending HIV as a public health challenge by 2030.

As part of the Pragmatic Trial for HIV Pre-Exposure Prophylaxis Roll-Out in Tanzania (PREPTA), an interactive and free-of-charge smartphone app (called *Jichunge*) was developed with the aim of supporting the initiation and use of PrEP among the following two groups, who are at increased HIV risk: female sex workers and men who have sex with men. In this paper, we evaluate the extent and predictors of retention in the *Jichunge* app among female sex workers after a duration of 6 months.

## Methods

### Study Design

This study was a prospective cohort analysis of participants who were recruited into the PREPTA study, which was partly described in our previous publications [17,18]. Briefly, the PREPTA is a research project with the overall aim of assessing the effectiveness of an mHealth intervention in promoting retention in PrEP care and adherence to PrEP among female sex workers and men who have sex with men in the following two cites of Tanzania: Dar es Salaam (intervention arm) and Tanga (control arm). The project is being implemented by the Muhimbili University of Health and Allied Sciences, Tanzania, and the University of Oslo, Norway. This study analyzed app usage after 6 months among female sex workers who were recruited in the intervention arm of the trial.

### Intervention

**Figure 1.**
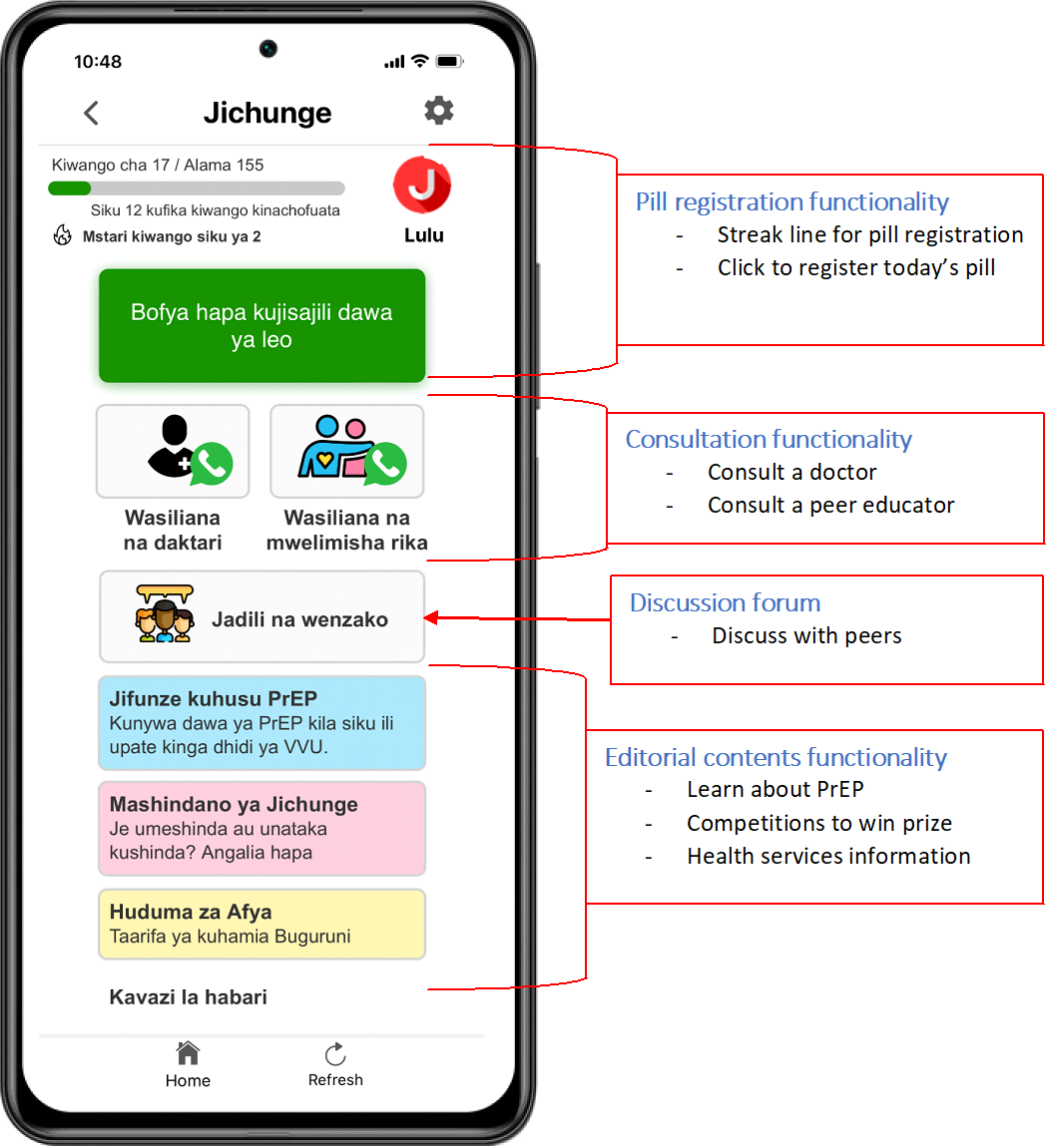
Main functionalities of the *Jichunge* app. PrEP: pre-exposure prophylaxis.

A participatory design approach was adopted when developing the *Jichunge* app [17]. The app provides users with information about PrEP, reminds them to take their daily pill, provides an opportunity to consult a doctor and a peer educator via the web, and operates a web-based forum for anonymous discussions among PrEP users (Figure 1). The overall aim of the app is to improve adherence to PrEP and retention in the PrEP program. Further details about the *Jichunge* app can be found in our previous publications [17,18].

### Study Population and Sample Size

We included women aged at least 18 years who had sold sex in the past 3 months. Participants were residents of Dar es Salaam (they had an address in the city and had lived there for the past 6 months), owned a smartphone, and were prepared to start PrEP treatment. The sample size for this analysis was estimated by using a formula for cohort studies [19,20]. Since the retention in PrEP services and adherence to PrEP were not known at the design stage of this study, we used a proportion (p) of 50%, which has been shown to provide an optimum sample size when the actual proportion is between 10% and 90% [21]. A precision of 5% and a design effect of 2 were used in estimating the sample size to achieve a statistical power of 80%. After adjusting for a 20% potential loss to follow-up, the minimum sample size was estimated to be 423 female sex workers.

### Study Procedures

Participants were recruited between March and June 2021, using respondent-driven sampling (RDS). RDS is a method for sampling from populations for which there is no available sampling frame [19,22]. On the first day, 3 initial participants (referred to as “seeds”) were recruited purposively by researchers and peer educators who had prior experience with biobehavioral surveys among female sex workers in Tanzania. The first seed was aged 20 years and was a street-based sex worker. The second seed was 32 years old and was a phone- and internet-based sex worker. The third seed was 28 years old and was a bar- and pub-based sex worker. Before the end of the recruitment phase, an additional 6 seeds were added to speed up recruitment, as individuals in the initial seeds' recruitment chains were facing difficulties in recruiting more participants. After having completed the study procedures, each seed was given 3 recruitment coupons and asked to pass them on to her peers and invite them to participate in the PREPTA study. Of the 9 seeds, only 1 did not recruit any other participant to the study. Every new study participant was asked to do the same until we had reached the desired sample size.

All participants were screened for PrEP eligibility by a health clinic, as per national guidelines [23]. PrEP eligibility criteria included a negative HIV test result, no signs of acute HIV infection, a serum creatinine clearance of >60 mL/min, and the willingness to start PrEP. Those who qualified were provided with PrEP pills for 30 days and thereafter invited to take part in the PREPTA study. After consenting to participate in the study, participants received the *Jichunge* app (free of charge), attended an app onboarding session, and thereafter participated in a face-to-face baseline interview with a trained interviewer. The *Jichunge* app was only available to study participants, and it was not available in mainstream app stores. If study participants needed to reinstall the app, they had to contact the project site managers and be given a link, through which they could download the app again. Upon completion of the interview, each participant was paid the modest amount of TZS 8000 (US $3.18) as compensation for transport costs and the time spent at the study site. In addition, participants received TZS 4000 (US $1.59) for each peer they referred to the study, as per the RDS protocol.

All participants were invited to participate in a 6-month follow-up interview regardless of whether they had used PrEP and the *Jichunge* app. For the 6-month follow-up data collection, we attempted to contact each participant by phone at least 3 times on different days before they were considered lost to follow-up.

### Data Collection

Information on sociodemographics and other structural factors at baseline was collected by using a questionnaire, which was administered by trained research assistants. At 6 months following enrollment, participants were invited to participate in a follow-up survey consisting of an interviewer-administered questionnaire that focused on their experiences with PrEP and the *Jichunge* app. From the back end of the app, we also continuously collected data on participants’ use of the app’s different functionalities (ie, opening the app, registering medicine taking, reading editorial contents, accessing a web-based consultation, and entering the web-based discussion forum). Data from the back end of the app were collected from all participants regardless of whether they participated in the 6-month follow-up survey. In this study, we included user statistics pertaining to participants' use of the *Jichunge* app after 6 months (ie, the 60-day period following the first 150 days of being in the intervention arm).

### Ethical Considerations

This study was conducted in accordance with the Declaration of Helsinki. Details on how we worked to protect participants’ privacy were provided in a previous article [18]. Briefly, data from the questionnaires were stored on a secure server that was developed particularly for the handling of sensitive research data (a service for sensitive data called “TSD” [24]) at the University of Oslo, Norway. The *Jichunge* app does not store, transfer, or expose sensitive data. Further, no element in the user interface of the app refers to any information that can identify a participant. Written informed consent was obtained from all participants involved in this study for the collection and use of both questionnaire data and app data. The protocol for this study was reviewed and approved by the National Health Research Ethics Committee in Tanzania (protocol code: NIMR/HQ/R.8a/Vol. IX/3454) and by the Regional Committee for Medical and Health Research Ethics in Norway (protocol reference number: 33675).

### Study Variables

#### Outcome Variable

The primary outcome variable for this study was 6-month retention in the *Jichunge* app. Retention in an mHealth app is one of the metrics for user engagement with mHealth apps [25-27]. In this study, participants were considered retained if they still used the app after 6 months. We assumed that after 6 months of intervention, a user would have established a routine and integrated the *Jichunge* app into their health care regimen. According to the PrEP implementation framework in Tanzania, a 6-month period is considered long enough to monitor a PrEP user for the effective use of PrEP and the presence of continuous HIV risk factors [23]. Accordingly, a 6-month period is long enough to evaluate user engagement with mHealth interventions that aim to promote the use of PrEP in the context of Tanzania. We defined *6-month retention* as app use within the 150 to 210 days following the first installation of the app, that is, within this 60-day period, one needed to open the app at least once to be categorized as “retained.”

#### Independent Variables

Independent variables were sociodemographics, sex work characteristics, and some sociostructural factors that were asked about at baseline and considered potentially associated with 6-month retention in the app. These included the age of the respondent, marital status, education level, social support, awareness of PrEP, experience with PrEP and sex work stigma, self-perceived HIV risk, and income from sex work. The measurement of these variables was done as described in the following paragraphs.

Social support was measured by using a Likert scale of 8 items, which was adapted from the Duke–UNC (University of North Carolina) Functional Social Support Questionnaire [28]. Participants were asked to respond to each question by choosing 1 of 5 possible responses (1=*Much less than I would like*; 2=*Less than I would like*; 3=*Some, but would like more*; 4=*Almost as much as I like*; 5=*As much as I like*). We computed the total score for all items, and a total score below 32 was considered to indicate “inadequate social support.” The scale had a Cronbach α of .88, indicating high internal consistency.

Sex work stigma and perceived PrEP stigma were measured by using 13 and 10 scale items, respectively; each item had 5 response options (1=*Strongly disagree*; 2=*Disagree*; 3=*Neither disagree nor agree*; 4=*Agree*; 5=*Strongly agree*), and the scales had a Cronbach α of .84 and .88 respectively, signifying high reliability. Sex work stigma was thereafter categorized into the following three categories: “low” for scores of ≤26, “moderate” for scores between 27 and 38, and “high” for scores of ≥39. For PrEP stigma, a score above 30 was considered “high.”

PrEP awareness was measured by using 8 true or false questions about PrEP. Participants who answered more than 6 questions correctly were categorized as being highly aware of PrEP.

### Statistical Analyses

We started by generating the distribution of sociodemographic characteristics and descriptive statistics related to the use of the *Jichunge* app. Using the chi-square test, we assessed the association of 6-month retention in the app with sociodemographics, sex work characteristics, and other sociostructural factors. Since the outcome variable was binary and relatively common (proportion of retained participants: 63/470, 13.4%), we used a generalized linear model with a log link and binomial distribution (log-binomial regression) to examine independent predictors of 6-month retention in the app [29]. Log-binomial regression was chosen because it estimates the risk ratios directly, as opposed to logistic regression, which gives odds ratios that can overestimate the association if the outcome variable is common (proportion of >10%) [30]. We started with a bivariate analysis to obtain crude estimates of risk ratios. All variables with a *P* value of <.25 in the bivariate analysis were included in the multivariable model to obtain adjusted estimates of risk ratios. All analyses were performed using Stata version 17 (StataCorp LLC), and a *P* value of <.05 was considered statistically significant.

## Results

### Sociodemographic Characteristics

A total of 470 participants (age: median 26, IQR 22-30 years) were recruited at baseline. Of these, 277 (58.9%) reported to have had at least some secondary education, and 361 (76.8%) had never been married. Out of all 470 participants, we managed to reach 340 (72.3%) participants for the 6-month follow-up survey, and 284 (60.4%) participants were interviewed.

### Participants’ Possession of the *Jichunge* App After 6 Months

Participants who came to the 6-month follow-up interview (n=284) were asked if they still had the *Jichunge* app on their phones; nearly three-quarters (206/284, 72.5%) no longer had the app. When asked about the reasons for this, a large majority of these 206 study participants (n=193, 93.7%) said that they no longer had access to the app because of issues related to their phones, such as the phone having been stolen or lost (n=89, 43.2%), the phone having been sold or changed (n=53, 25.7%), or the phone having technical problems (n=51, 24.8%); only a few (n=13, 6.3%) reported to have uninstalled the app from their phones.

### Pattern of the Use of the *Jichunge* App and Its Services

**Figure 2.**
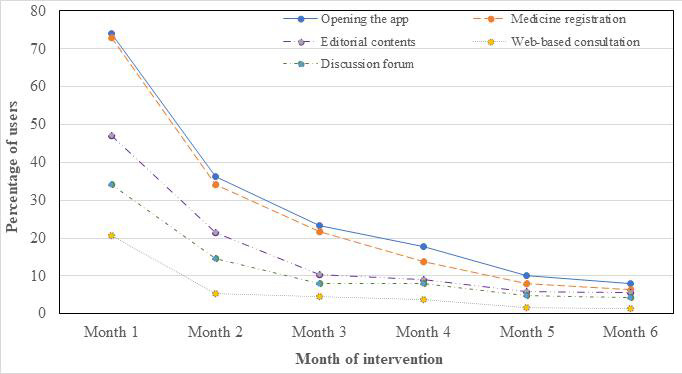
Pattern of use of the *Jichunge* app and its services.

More than three-quarters of the participants (376/470, 80%) had accessed the app at least once after enrollment into the PREPTA study. Figure 2 shows the proportion of study participants who had opened the app and used different *Jichunge* services at least once per month during the 6-month observation period. App use was high in the first month and declined continually in the subsequent months. For all months, pill registration was the most used service, while web-based consultation was the least used service.

### Retention in the *Jichunge* App and Use of Its Services After 6 Months

Overall, of the 470 participants, 63 (13.4%) were retained in the sense that they had opened the app at least once after 6 months of enrollment. Specifically, of the 470 participants, 52 (11.1%) had registered their daily pill taking, 40 (8.5%) had accessed PrEP editorial contents, 35 (7.4%) had used the discussion forum, and 14 (3%) had used the *Jichunge* web-based consultation services.

### Participants’ Baseline Characteristics by 6-Month Retention in the *Jichunge* App

In Table 1, we present the distribution of participants’ baseline characteristics by retention in the *Jichunge* app at 6 months. Retention increased significantly and linearly with age; 10.6% (21/199) of women aged 18 to 24 years were retained, whereas 12.8% (29/226) of women aged 25 to 34 years and 28.9% (13/45) of women aged ≥35 years were retained (*χ*^2^_2_=10.8; *P*=.01). The proportion of retained users was also higher among women with high PrEP awareness (41/232, 17.7%) than that among women with low PrEP awareness (22/238, 9.2%; *χ*^2^_1_=7.2; *P*=.01). Retention was higher among female sex workers who had experienced financial difficulties due to health care spending (43/244, 17.6%) than that among female sex workers who had not experienced such difficulties (19/224, 8.5%; *χ*^2^_1_=8.2; *P*=.004).

**Table 1.** Distribution of participants’ baseline characteristics by 6-month retention in the *Jichunge* app (N=470).

Characteristics		All participants, n (%)	Participants retained, n (%)	Participants not retained, n (%)	Chi-square (*df*)	*P* value
**Age (years)**					10.8 (2)	.01^a^
	18-24	199 (42.3)	21 (10.6)	178 (89.4)		
	25-34	226 (48.1)	29 (12.8)	197 (87.2)		
	≥35	45 (9.6)	13 (28.9)	32 (71.1)		
**Marital status**					0.02 (1)	.90
	Never married	361 (76.8)	48 (13.3)	313 (86.7)		
	Married or previously married	109 (23.2)	15 (13.8)	94 (86.2)		
**Education**					2.3 (2)	.32
	Formal education	28 (6)	5 (17.9)	23 (82.1)		
	Primary education	165 (35.1)	17 (10.3)	148 (89.7)		
	Secondary education or higher	277 (58.9)	41 (14.8)	236 (85.2)		
**Social support^b^**					2.6 (1)	.11
	Inadequate	283 (60.2)	44 (15.6)	239 (84.4)		
	Adequate	184 (39.1)	19 (10.3)	165 (89.7)		
**PrEP^c^ awareness**					7.2 (1)	.01
	Low	238 (50.6)	22 (9.2)	216 (90.8)		
	High	232 (49.4)	41 (17.7)	191 (82.3)		
**Financial difficulties due to health care spending**					8.2 (1)	.004
	Yes	244 (51.9)	43 (17.6)	201 (82.4)		
	No	224 (47.7)	19 (8.5)	205 (91.5)		
**Sex work stigma^b^**					0.5 (2)	.79
	Low	27 (5.7)	4 (14.8)	23 (85.2)		
	Moderate	398 (84.7)	53 (13.3)	345 (88.7)		
	High	32 (6.8)	3 (9.4)	29 (90.6)		
**Perceived PrEP stigma^b^**					0.3 (1)	.59
	Low	355 (75.5)	46 (13)	209 (87)		
	High	114 (24.3)	17 (14.9)	97 (85.1)		
**Income from sex work (TZS [US $])^b^**					2.4 (3)	.49
	≤150,000 (≤59.61)	116 (24.7)	15 (12.9)	101 (87.1)		
	150,001-299,999 (59.61-119.25)	108 (23)	13 (12)	87 (88)		
	300,000-449,999 (119.22-178.87)	144 (30.6)	25 (17.4)	119 (82.4)		
	≥450,000 (≥178.82)	90 (19.1)	10 (11.1)	80 (86.2)		

a *P* value based on the chi-square trend test.

b N is less than 470 due to missing observations.

c PrEP: pre-exposure prophylaxis.

### Predictors of 6-Month Retention in the *Jichunge* App

Table 2 shows the results of the log-binomial regression of the independent predictors of retention in the *Jichunge* app. The findings revealed that older age, high PrEP awareness, and financial difficulties due to health care spending independently predicted retention in the app. Participants aged at least 35 years were more than 2 times more likely to be retained in the app than those aged 18 to 24 years (adjusted risk ratio [aRR] 2.3, 95% CI 1.2-4.1; *P*=.01). Similarly, participants who demonstrated high PrEP awareness at baseline were more likely to be retained in the app after 6 months than those who demonstrated low PrEP awareness (aRR 1.8, 95% CI 1.1-3; *P*=.01). Furthermore, female sex workers who had experienced financial difficulties due to health care spending were approximately 2 times more likely to be retained in the app after 6 months than those who had not experienced such difficulties (aRR 1.9, 95% CI 1.2-3.2; *P*=.01).

**Table 2.** Log-binomial regression model for independent predictors of 6-month retention in the *Jichunge* app.

Variable		Risk ratio (95% CI)	*P* value	Adjusted risk ratio (95% CI)	*P* value
**Age (years)**					
	18-24	1	Reference	1	Reference
	25-34	1.2 (0.7-2.1)	.47	1.1 (0.7-1.9)	.64
	≥35	2.74 (1.5-5)	.001	2.2 (1.2-4.1)	.01
**Social support**					
	Adequate	1	Reference	1	Reference
	Inadequate	1.5 (0.9-2.6)	.12	1.4 (0.8-2.3)	.23
**Financial difficulties due to health care spending**					
	Yes	2.1 (1.2-3.4)	.01	1.9 (1.2-3.2)	.01
	No	1	Reference	1	Reference
**PrEP^a^ awareness**					
	High	1.9 (1.2-3.1)	.01	1.8 (1.1-3)	.01
	Low	1	Reference	1	Reference

a PrEP: pre-exposure prophylaxis.

## Discussion

### Principal Findings

Although several mHealth interventions have been designed to support the delivery of health care services, few studies have evaluated retention in those interventions. Understanding the retention in mHealth apps is crucial when planning and designing interventions. In this study, we examined the extent and predictors of 6-month retention in an interactive mHealth app (*Jichunge* app) designed to promote adherence to PrEP use and retention in PrEP care within Tanzania, a lower-middle–income country in sub-Saharan Africa.

The proportion of retained female sex workers (those who still used the *Jichunge* app after 6 months) was only 13.4% (63/470). This is much lower than the the optimal 46.4% (218/470) of users we found 1 month after the app had been installed [18]. The main reason for the lower retention in the app after 6 months did not appear to have much to do with the app itself but rather with the fact that a very large proportion of users had lost access to the app due to their phones having been lost, stolen, sold, changed, or damaged. Issues related to phones clearly represented a main challenge for implementing this mHealth intervention among female sex workers in Tanzania.

We have not found any previous study that evaluated the extent of retention in mHealth apps for HIV prevention. However, our results are in line with those of a study conducted in Poland, which found that less than one-quarter of participants used mHealth to monitor diet, weight, and physical activity in 2022 [31]. On the other hand, our estimate was lower than those reported in studies of the engagement with mHealth apps for self-management among patients with cancer in Sweden [8] and among adolescent and young adult cancer survivors in Pennsylvania [32]. It was also lower than the engagement with text message–delivered support among patients with type 2 diabetes in Tennessee [9]. The difference in the extent of use could be due to variations in contextual, population, and intended health outcomes for each of the interventions. Importantly, the studies mentioned for comparison entailed people diagnosed with chronic medical conditions, while PrEP is used for prevention among healthy people; therefore, the motivation to use the app might be different. Thus, when designing mHealth apps, strategies for attracting users and promoting long-term retention should take into consideration the contextual setting and the specific needs of the targeted population.

Understanding predictors of retention in mHealth apps is essential in tailoring the design of mHealth for its intended users. In this study, we found that retention in the *Jichunge* app was associated with older age, high PrEP awareness, and financial difficulties due to health care spending. Contrary to the evaluation of the use of the *Jichunge* app after 1 month [18], education level and social support were not associated with the retention in the app. This implies that the predictors of early mHealth use are not necessarily the same as those for retention in mHealth apps. Thus, continual evaluation of mHealth interventions is essential in understanding the users’ early use and long-term retention behaviors.

In this study, older female sex workers were more likely to be retained in the *Jichunge* app than younger women after 6 months. Our findings are similar to those in a study by Chang et al [33], who found that older persons were more likely to use mobile phones for opioid use disorder telemedication during the COVID-19 pandemic in New York. Contrary to this, Bauer et al [34] found that younger age was associated with the use of mHealth tools among primary care patients in the Northwest United States. The use of the *Jichunge* app does not require sophisticated technical skills, and this might have contributed to easier use among older women. On the other hand, the app does not contain entertaining features, such as those found in many commercial social media apps, and this might have discouraged younger persons from engaging with the app over time. Therefore, future mHealth app development may consider the inclusion of features that may speak to the different needs of younger and older populations.

Participants who demonstrated high PrEP awareness at baseline were more likely to be retained in the *Jichunge* app after 6 months. A lack of prior awareness of medication is among factors that can affect the willingness to use PrEP and hence can affect the acceptability and use of a PrEP-related app [35]. Therefore, people with prior awareness of PrEP medication might have been motivated to use the *Jichunge* app to learn more about the medication. The primary goal of *Jichunge* was to promote adherence to PrEP and retention in PrEP care; hence, it is likely that participants who stopped using PrEP might have lost interest in the *Jichunge* app as well. The findings underline the need to create awareness about PrEP before launching innovative interventions to support PrEP adherence and retention.

One of the benefits of many mHealth interventions is that they can reduce the financial costs related to health care services [36,37]. In this study, we found that participants who reported to have experienced financial difficulties due to health care spending were more likely to be retained in the *Jichunge* app than those who did not have such difficulties. In Tanzania, PrEP is provided free of cost, and at the time of this study, only a few clinics were designated to prescribe PrEP. Participants with financial challenges could have been using *Jichunge* services, such as the web-based consultation that is provided free of cost, as an alternative to physical consultations for PrEP-related and other concerns or conditions. Our findings are contrary to those reported in a retrospective comparative study conducted in Boston by Xiong et al [38], who found that patients insured through Medicaid were less likely to use telemedicine to connect with orthopedic surgery services than privately insured patients. On the basis of our study, mHealth apps seem to have the potential to help improve universal health coverage for underserved populations by removing some of the financial barriers associated with access to health care services.

### Strength and Limitations

First, among the strengths of this study is that it provides evidence on retention in mHealth apps within a real-world setting. Second, the large sample size ensured enough statistical power and stable estimates. Third, we evaluated the outcome variable (6-month retention) by using objective measures (electronic records of actual app use), thereby avoiding the bias that could have resulted from using subjective measures (such as self-reported use of the app). One limitation of this study is that we did not carry out any in-depth investigation of users’ experiences with the *Jichunge* app, which limits our understanding of why our participants did or did not engage with the mHealth intervention. Furthermore, we only measured 1 aspect of user engagement with the app (retention), which limits our understanding of other aspects, such as time spent in the app, total sessions, and subjective measures.

### Conclusion

Overall, retention in the *Jichunge* app after 6 months was low and was predicted by older age, high PrEP awareness, and financial difficulties due to health care spending. A large majority of the sex workers (193/206, 93.7%) who took part in the 6-month follow-up interview reported that they no longer had access to the *Jichunge* app because their phones had been lost, sold, stolen, or changed or were in disrepair. Clearly, female sex workers in Tanzania cannot be expected to use the same phone over long periods of time, and this may underscore some of the complexities associated with mHealth interventions in this population. Thus, the user patterns and “social lives” of smartphones should be taken into consideration when designing mHealth interventions. We recommend qualitative studies that could provide in-depth information on the users’ experiences with the use of mHealth interventions for PrEP services.

## Abbreviations

aRR: adjusted risk ratio
mHealth: mobile health
PrEP: pre-exposure prophylaxis
PREPTA: Pragmatic Trial for HIV Pre-Exposure Prophylaxis Roll-Out in Tanzania
RDS: respondent-driven sampling
UNAIDS: Joint United Nations Programme on HIV/AIDS
UNC: University of North Carolina
